# Developing and validating an algorithm to identify incident chronic dialysis patients using administrative data

**DOI:** 10.1186/s12911-020-01206-x

**Published:** 2020-08-11

**Authors:** Dino Gibertoni, Claudio Voci, Marica Iommi, Benedetta D’Ercole, Marcora Mandreoli, Antonio Santoro, Elena Mancini

**Affiliations:** 1 Department of Biomedical and Neuromotor Sciences, University of Bologna, Via San Giacomo 12, 40126 Bologna, Italy; 2 Bologna Local Health Authority, Bologna, Italy; 3 Advanced School for Health Policy, University of Bologna, Bologna, Italy; 4 Cooperativa EDP La Traccia, Matera, Italy; 5 Nephrology and Dialysis Unit, S. Maria della Scaletta Hospital, Imola, Italy; 6 Specialty School in Nephrology, University of Bologna, Bologna, Italy; 7 Nephrology, Dialysis and Hypertension Unit, Policlinico S.Orsola-Malpighi, Bologna, Italy

**Keywords:** Chronic dialysis, Administrative data, Hospital discharge records, Ambulatory specialty visits, Case definition, Algorithm

## Abstract

**Background:**

Administrative healthcare databases are widespread and are often standardized with regard to their content and data coding, thus they can be used also as data sources for surveillance and epidemiological research. Chronic dialysis requires patients to frequently access hospital and clinic services, causing a heavy burden to healthcare providers. This also means that these patients are routinely tracked on administrative databases, yet very few case definitions for their identification are currently available. The aim of this study was to develop two algorithms derived from administrative data for identifying incident chronic dialysis patients and test their validity compared to the reference standard of the regional dialysis registry.

**Methods:**

The algorithms are based on data retrieved from hospital discharge records (HDR) and ambulatory specialty visits (ASV) to identify incident chronic dialysis patients in an Italian region. Subjects are included if they have at least one event in the HDR or ASV databases based on the ICD9-CM dialysis-related diagnosis or procedure codes in the study period. Exclusion criteria comprise non-residents, prevalent cases, or patients undergoing temporary dialysis, and are evaluated only on ASV data by the first algorithm, on both ASV and HDR data by the second algorithm. We validated the algorithms against the Emilia-Romagna regional dialysis registry by searching for incident patients in 2014 and performed sensitivity analyses by modifying the criteria to define temporary dialysis.

**Results:**

Algorithm 1 identified 680 patients and algorithm 2 identified 676 initiating dialysis in 2014, compared to 625 patients included in the regional dialysis registry. Sensitivity for the two algorithms was respectively 90.8 and 88.4%, positive predictive value 84.0 and 82.0%, and percentage agreement was 77.4 and 74.1%.

**Conclusions:**

Algorithms relying on retrieval of administrative records have high sensitivity and positive predictive value for the identification of incident chronic dialysis patients. Algorithm 1, which showed the higher accuracy and has a simpler case definition, can be used in place of regional dialysis registries when they are not present or sufficiently developed in a region, or to improve the accuracy and timeliness of existing registries.

## Background

The prevalence of end stage kidney disease (ESKD) requiring dialytic treatment is growing worldwide, due to population ageing and the increased prevalence of comorbidities [1–3]. Patients on dialysis have a high mortality risk [4–6] and their management is crucial for health care providers, because of the high organizational and financial burden related to the frequency and the complexity of the treatment [7–10]. Therefore, it is extremely important to implement population-based registries of patients in dialysis, to assist health care providers in organizing the treatment and to have data available to evaluate the patients’ health outcomes. Many national or regional registries of patients on dialysis or on renal replacement therapy (RRT) have been created since the 1990s; as a result, in Europe the ERA-EDTA Registry currently gathers information from more than 50 countries and regions and produces yearly a detailed report [11]. However, to implement and regularly maintain a regional dialysis registry a large amount of dedicated human resources would be needed. This is actually true in Italy, that conferred data from only 8 out of 21 regions for the last published ERA-EDTA registry report [11]. One way to overcome this problem is to exploit the data recorded in the official administrative databases and create a regional registry by means of an automated algorithm. Very few algorithms and case definitions for incident dialysis patients can be found in the literature: Clement et al. [12] presented and validated a case definition based on Canadian outpatient physician billings, and other algorithms to identify RRT patients from administrative data were proposed using data from Australian and Italian regions [13, 14]. In this study, we submit a novel algorithm that identifies incident dialysis patients taking advantage of data from the hospital discharges and outpatient specialty databases. We validated the algorithm by comparison with the gold standard represented by the dialysis registry of the Emilia-Romagna region. This algorithm relies on ICD9-CM coding and can be easily adapted to be utilized in other region and countries, where it could aid to implement a new registry of chronic dialysis patients or improve the quality of existing registries.

## Methods

### Algorithms

We describe two algorithms designed to identify the incident chronic dialysis patients in a selected time period (the *index period*) using data obtained from the regional hospital discharge records (HDR) and ambulatory specialty visits (ASV) databases. The algorithms require all dialysis events recorded from 1 year before the starting date to 1 year after the ending date of the index period. In this study, we tested the algorithms by identifying the incident chronic dialysis patients in 2014, thus we built a dataset containing hospital admissions and ambulatory specialty visits from 1.1.2013 to 31.12.2015. The two algorithms used the same inclusion criteria:

- in HDR, at least one admission in the index period with ICD9-CM main or secondary diagnosis codes 585.6 (end stage renal disease, excluding admissions aimed to create the arteriovenous or peritoneal fistula, identified by 39.27, 39.29 or 54.93 codes as the only dialysis-related procedure code), V45.1 (renal dialysis status), or V56.0-V56.8 (dialysis encounter). In this case, the admission date was used to set the dialysis date;
- or, in HDR, at least one admission with any procedure code ICD9-CM 39.95 (hemodialysis) or 54.98 (peritoneal dialysis) In this case, the date of the procedure was used to set the dialysis date; when more than one dialysis procedure was found, the earliest date was used. If the HDR record included both diagnosis and procedure codes related to dialysis, the procedure date was selected;
- or, in ASV, at least one hemodialysis (codes 39.95.1–39.95.9) or at least one peritoneal dialysis (codes 54.98.1 or 54.98.2) in the index year.

For each patient, the date of the first dialysis (*index date*) was defined as the earliest among the dates of visits and hospital admissions of 2014.

Exclusion criteria were:

- patients not residing in the Emilia-Romagna region: they were excluded if at least one record from the HDR and ASV databases in the index period indicated a different region of residence;
- prevalent cases: patients were excluded if at least one hemodialysis or peritoneal dialysis was found in the 365 days before the index date;
- non-chronic dialysis patients: patients were excluded if, within 1 year following the index date, less than 30 days passed between the index date and the last dialysis date, or less than 90 days for patients who initiated dialysis for acute kidney injury (AKI, primary or secondary diagnosis code ICD9-CM 584.x found in the index dialysis admission or in admissions occurred less than 90 days before the index date). This criterion allows to exclude patients who died, or recovered, or were transferred to a nephrology unit of another region shortly after dialysis initiation;
- patients who died during the hospitalization in which the first dialytic treatment was provided. Only algorithm 2 used this criterion.

To facilitate algorithms’ implementation for users whose data are classified according to ICD-10-CM classification, a conversion table of the required codes is provided (Table 1). The two algorithms search for the index dialysis in both HDR and ASV databases, but differ in the definition of chronic dialysis: algorithm 1 defines as chronic dialysis patients those who are seen regularly in outpatient clinics, and therefore searches for previous and subsequent dialysis events only in the ASV database. Algorithm 2 considers also dialysis treatments provided during hospitalizations, and thus uses data from both the ASV and the HDR databases. A variation of algorithm 1 in which the index date is retrieved only from ASV records was also tested, to address situations in which only ASV data are available. Sensitivity analyses were performed for both algorithms by using 60 and 90 days after the index date as thresholds to define non-chronic dialysis patients. The algorithms were developed in Stata 15.1 and SAS Enterprise Guide 7.1.

**Table 1:** Conversion of ICD-9-CM codes used by the algorithms in ICD-10-CM codes (2020 edition)

ICD-9-CM codes	ICD-9-CM descriptions	ICD-10-CM codes	ICD-10-CM Descriptions
584	Acute renal failure	N17	Acute renal failure
585.6	End stage renal disease	N18.6	End stage renal disease
V45.11	Renal dialysis status	Z99.2	Dependence on renal dialysis
V45.12	Noncompliance with renal dialysis	Z91.15	Patient’s noncompliance with renal dialysis
V56.0	Encounter for extracorporeal dialysis	Z49.31	Encounter for adequacy testing for hemodialysis
V56.1	Fitting and adjustment of extracorporeal dialysis catheter	Z49.01	Encounter for fit/adjst of extracorporeal dialysis catheter
V56.2	Fitting and adjustment of peritoneal dialysis catheter	Z49.02	Encounter for fit/adjst of peritoneal dialysis catheter
V56.31	Encounter for adequacy testing for hemodialysis	Z49.31	Encounter for adequacy testing for hemodialysis
V56.32	Encounter for adequacy testing for peritoneal dialysis	Z49.32	Encounter for adequacy testing for peritoneal dialysis
V56.8	Encounter for other dialysis	Z49.32	Encounter for adequacy testing for peritoneal dialysis

### Accuracy of algorithms

The algorithms were tested on data taken from the administrative databases of Emilia-Romagna, a region in north-western Italy with 11 local health authorities in 2014 (currently merged into 8) serving a population of 4.446 million inhabitants as of 1/1/2014 (data provided by Istat at https://www.istat.it/en/population-and-households?data-and-indicators). According to the regional dialysis registry, in 2014 the number of incident dialysis patients in Emilia-Romagna was 685, corresponding to 154 per million population (pmp), compared to 159 pmp in Italy [15].

Records from the Emilia-Romagna regional administrative databases were linked using a unique pseudonymized patient identifier, which allows capturing all patient encounters with the health care system. The data taken from the HDR and ASV databases to feed the algorithms belong to a subset of compulsory data homogeneously recorded in all Italian regions, as they are an information obligation to the Ministry of Health. Specifically, the hospital discharge records database contains admission date, discharge date, intervention date, specialty at discharge, up to 15 diagnostic codes and 15 procedure codes [International Classification of Disease (ICD-9 CM)] for each admission. The ASV database contains records regarding each hemodialysis session or one monthly summary record for peritoneal dialysis; in both cases, data on the type of service delivered (laboratory test, specialty service, rehabilitation service), the medical discipline related to the service, the date of delivery are present. Due to the current Italian regulation on privacy, we could not use the regional patient registry to obtain patients’ personal data, thus the residence of patients was ascertained from the data present in the HDR and ASV databases.

### Gold standard

The Emilia-Romagna regional Dialysis Registry (ERDR) was used as the reference source to evaluate the accuracy of the two algorithms. The ERDR was established in 1994 and is part of the Italian Dialysis Registry network, which in turn feeds the ERA-EDTA Dialysis Registry; as such it is the official source of epidemiological information about chronic dialysis in the Emilia-Romagna region. Incident cases in the ERDR are patients living in the Emilia-Romagna region who initiate dialysis for the first time according to the intention-to-treat approach. The dialysis inception date reported in the ERDR is assigned by the nephrologist in charge, and is the date in which the patient started being considered as chronic. Patients who stop dialysis because of transplantation are maintained in the registry as “transplanted patients” (hence, they are not included in the population of prevalent dialysis patients) and if they subsequently reinitiate dialysis, they will be considered as new entries for that year. Patients starting dialysis following an AKI and not chronicised, as well as guests (patients temporarily treated with dialysis in a regional clinic but living elsewhere, in Italy or abroad) are not added to the ERDR. The registry is updated yearly by transferring data from the information systems of nephrology units, checked for data quality by the nephrologist in charge of the registry (EM) and uploaded on a dedicated website (https://www.regdial.it). In 2014 all but one of the local nephrology units could automatically transfer data from their patients’ management information systems to the ERDR, therefore widely reducing the possible mistakes due to manual data transcription. However, the ERDR is regularly monitored and revised for inconsistencies and missing data.

The comparison between the algorithms and the ERDR was made by calculating only percentage agreement, sensitivity and positive predictive value (PPV), because the ERDR does not contain subjects without dialysis.

## Results

Overall 610,724 records of 6108 patients with at least one dialysis event in 2014 were retrieved from HDR and ASV. From these, data provided by two local health authorities were excluded from the analysis because their ASV records data for the years 2013–14 were largely incomplete. After applying exclusion criteria, 680 incident chronic dialysis patients were identified by algorithm 1 and 676 by algorithm 2. The patients mutually identified by both algorithms were 631. The ERDR included 625 incident patients in 2014 after removing those treated in the two local health authorities with incomplete data. Cases recorded in the ERDR and missed by the algorithms were respectively 58 and 72. Algorithm 1 showed sensitivity = 90.8% and PPV = 84.0% (Table 2) and algorithm 2 had a slightly lower performance (sensitivity =88.5% and PPV = 82.0%). Algorithm 2 was less accurate mostly because it identified more chronic incident in dialysis patients unknown to ERDR (68 vs. 39) on the basis of occasional hospital admissions while not having had any dialysis related specialty visit in the year following the index date. The estimated incidence rate of dialysis inception per million regional population was 168 for ERDR, 182 for algorithm 1 and 181 for algorithm 2. The algorithm that used only ASV data was very similar to algorithm 1 in terms of PPV (83.8%) and inferior for agreement and sensitivity (Table 3). Sensitivity analyses confirmed that Algorithm 1 with threshold for chronicity at 30 days provided the best performance; all algorithms’ variations displayed very good sensitivity, while PPV generally was around 5% lower.

**Table 2:** Accuracy of the two algorithms vs. the Emilia-Romagna Dialysis (ERDR) registry

	Algorithm 1	Algorithm 2
Incident cases identified by the algorithm	680	676
found in ERDR, incident in 2014	571	554
found in ERDR, incident in a different year	70	54
not found in ERDR	39	68
Incident ERDR cases in 2014 missed by the algorithm	58	72
Percentage agreement	77.4%	74.1%
Sensitivity	90.8%	88.5%
Positive predictive value (PPV)	84.0%	82.0%

**Table 3:** Accuracy of the algorithms variants vs. the Emilia-Romagna Dialysis (ERDR) registry

	Algorithm using only ASV data	Algorithm 1 with 60 days’ threshold	Algorithm 1 with 90 days’ threshold	Algorithm 2 with 60 days’ threshold	Algorithm 2 with 90 days’ threshold
Incident cases identified by the algorithm	637	697	683	711	690
Found in ERDR, incident in 2014	534	550	542	538	531
Found in ERDR, incident in a different year	55	67	67	51	49
Not found in ERDR	48	80	74	122	110
Incident ERDR cases in 2014 missed by the algorithm	88	75	82	84	90
Percentage agreement	73.7%	71.2%	70.8%	67.7%	68.1%
Sensitivity	85.9%	88.0%	86.9%	86.5%	85.5%
Positive predictive value (PPV)	83.8%	78.9%	79.4%	75.7%	77.0%

The socio-demographic and clinical characteristics of the cohorts identified by the algorithms and those included in ERDR were overall very similar (Table 4): algorithm 1’s cohort was slightly younger than the ERDR’s cohort, and as such had a lower HD type of dialysis, lower number of treatments and less comorbidities.

**Table 4:** Characteristics of the cohorts identified by algorithms and by the regional dialysis registry

	Algorithm 1	Algorithm 2	ERDR
N	680	676	625
Age, mean ± sd	67.6 ± 15.0	67.3 ± 15.3	68.0 ± 14.6
Females, n (%)	233 (34.3)	236 (34.9)	215 (34.7)
Haemodialysis, n (%)	591 (86.9)	591 (87.4)	546 (88.2)
n. of treatments in the first year, median (p25-p75)	24 (11–103)	28 (11–105)	26 (11–106)
n. of hospital admissions in the first year, mean ± sd	2.6 ± 2.2	2.7 ± 2.2	2.1 ± 1.7
Charlson Comorbidity Index, median (p25-p75)	1 (0–3)	2 (1–3)	2 (1–3)

The main reasons for lack of agreement between the algorithms and ERDR were: a) discordant information on patients’ region of residence; b) patients recorded with different index dates, falling in two adjacent calendar years. For instance, a patient could be identified as prevalent by the algorithm on the basis of the treatment he received during a hospital admission in December 2013, while in the ERDR it was recorded as incident in January 2014; c) patients included in the ERDR although they were treated for less than 30 days (or 90 days in the case of AKI at the index dialysis), because of death, functional recovery or relocation to another Italian region or abroad.

## Discussion

The two algorithms developed in this paper showed good accuracy in the identification of incident chronic dialysis patients from routinely collected administrative data. Both algorithms are easy to implement because they need as input data taken from the hospital discharge records and outpatient specialty visits databases, which are implemented in many countries, and apply inclusion and exclusion criteria based on ICD-9 CM codes. Algorithm 1 can be preferred because it displayed the highest accuracy compared to the dialysis registry (90.8% sensitivity, 84.0% PPV) and it is based on a simpler case definition.

The accuracy of our algorithm is consistent to the findings of other case definitions of chronic dialysis that were elaborated in Canada [12, 16]. As these authors pointed out, the accuracy of their case definitions increased as the time interval on which they were applied was extended from 1 year to 5 years, thus we deem that the accuracy of our algorithm might also improve when searching for incident patients on a multi-year period. Actually, one of the reasons of mismatch between our algorithms and the ERDR reference is the date of dialysis inception recorded in those sources for the same patient, which may differ slightly and fall in two different calendar years. Clearly, this kind of discrepancy becomes less influent as the time period for incident patients’ identification is extended. Another relevant reason of mismatch was the inconsistency between the reported regions of residence. In regions with high migration rates there could actually be a non-negligible number of patients who change their residence near the dialysis inception and whose residency may be erroneously coded. However, this bias may be somewhat inflated in our validation, because we obtained data on residence from the ASV and HDR records which are less accurate than the regional registry of patients. There are also patients who experienced a complex therapeutic trajectory, alternating periods of dialysis treatment and periods of conservative treatment, which can make it difficult for an automated algorithm to discriminate whether they are under chronic or temporary treatment. Lastly, inaccuracies in the ERDR may be present, because the information on incident cases is added and checked manually. While this only marginally affected the algorithms’ performance, it means that an automated algorithm may also be used in parallel with an established registry and help improve its accuracy.

One peculiar feature of our algorithm is having established at 30 days after the index date the time needed to determine whether a patient initiating dialysis is chronic, in contrast to the more conventional minimum duration of 90 days [17]. We think that patients who underwent treatment for 30 to 90 days are worthy being considered as receiving chronic dialysis treatment, as they needed a relevant amount of resources, generated a non-negligible cost to the institution in which they were treated and may experience a renal outcome in that timeframe. Thus, we deem our case definition is more accurate to identify cohorts of patients to be considered in studies aimed to estimate the costs of dialysis treatment and, more generally, for epidemiological studies and outcome studies, taking advantage of the possibility to link the patients’ records to the other administrative databases.

An efficient case definition algorithm may also be useful to automatically feed a regional registry of dialysis patients, especially in those regions in which a registry is not yet established. Data from administrative databases have the advantage over manually fed data of being population-based and less error-prone [18], provided of course that the data source is of good quality. However, algorithms like those we developed may require specific adjustments of their parameters, depending on local characteristics. We found algorithm 1 with 30 days threshold was the best performant, but this should be confirmed by replication of the algorithm on different databases. The main limitation of an automated algorithm lies indeed in its dependence on the scope and quality of the administrative data, that are usually collected for purposes different from research. Thus, the implementation of an algorithm might also be a trigger for the identification of inaccuracy areas in the administrative data and lead to intervention aimed to improve the quality of data. Another limitation relates to the index date definition, which necessarily corresponds to an event recorded in the administrative data; the actual date from which the patient could not survive without dialysis may be different, especially when dialysis initiation was not programmed but followed an AKI. When such a difference exists it is usually slight, yet it could determine a small percentage of cases to be defined as incident in different years. Lastly, complex cases may not satisfy the criteria defined by the algorithm and require the experience of nephrologists to be adequately classified.

## Conclusions

We provide two algorithms that identify with good accuracy incident chronic dialysis patients from the administrative databases of hospital discharges and outpatient visits. The algorithm that identifies patients as chronic dialysis incidents if they attended ambulatory specialty visits after the first dialysis is to be preferred, because it is more accurate. These algorithms may be useful to create regional registries of chronic dialysis patients or to improve the accuracy of existing registries. As they are derived from administrative databases using ICD9-CM codes, with the necessary adaptations they can be used in many regional or national settings and can easily be linked to other data sources, representing a valuable tool for clinical and epidemiological studies. The performance of our algorithms should be further validated using data from other regions.

## Data Availability

The datasets generated and/or analysed during the current study are not publicly available due to the current regulation on privacy but are available from the corresponding author on reasonable request.

## Abbreviations

HDR: Hospital discharge records
ASV: Ambulatory specialty visits
ERDR: Emilia-Romagna dialysis registry
ESKD: End stage kidney disease
RRT: Renal replacement therapy
AKI: Acute kidney injury

## References

[1] Go AS, Chertow GM, Fan D, Mcculloch CE, Hsu C (2004). Chronic kidney disease and the risks of death, cardiovascular events, and hospitalization. N Engl J Med.

[2] De Lissovoy G (2007). Measuring the burden of illness for end-stage renal disease: some heavy lifting required. Kidney Int.

[3] Tonelli M, Wiebe N, Guthrie B, James MT, Quan H, Fortin M (2015). Comorbidity as a driver of adverse outcomes in people with chronic kidney disease. Kidney Int.

[4] Tonelli M, Wiebe N, Culleton B, Rabbat C, Fok M, House A (2006). Chronic kidney disease and mortality risk: a systematic review. J Am Soc Nephrol.

[5] Nordio M, Limido A, Maggiore U, Nichelatti M, Postorino M, Quintaliani G (2012). Survival in patients treated by long-term dialysis compared with the general population. Am J Kidney Dis.

[6] United States Renal Data System. 2018 USRDS annual data report: Epidemiology of kidney disease in the United States. Bethesda; 2018.. 10.1053/j.ajkd.2019.01.001 PMC6620109 30798791

[7] Levey AS, Atkins R, Coresh J, Cohen EP, Collins AJ, Eckardt KU (2007). Chronic kidney disease as a global public health problem: approaches and initiatives - a position statement from kidney disease improving global outcomes. Kidney Int.

[8] Pontoriero G, Pozzoni P, Del Vecchio L, Locatelli F (2007). International study of health care organization and financing for renal replacement therapy in Italy: an evolving reality. Int J Health Care Finance Econ.

[9] Roggeri A, Roggeri DP, Zocchetti C, Bersani M, Conte F (2017). Healthcare costs of the progression of chronic kidney disease and different dialysis techniques estimated through administrative database analysis. J Nephrol.

[10] Roggeri DP, Roggeri A, Salomone M (2014). Chronic kidney disease: evolution of healthcare costs and resource consumption from Predialysis to Dialysis in Piedmont region. Italy Adv Nephrol.

[11] Registry ERA-EDTA. ERA-EDTA Registry Annual Report 2017. Amsterdam; 2019.

[12] Clement FM, James MT, Chin R, Klarenbach SW, Manns BJ, Quinn RR (2011). Validation of a case definition to define chronic dialysis using outpatient administrative data. BMC Med Res Methodol.

[13] Brameld KJ, Thomas MAB, Holman CDAJ, Bass AJ, Rouse IL (1999). Validation of linked administrative data on end-stage renal failure: Application of record linkage to a “clinical base population.”. Aust N Z J Public Health.

[14] Valent F, Busolin A, Boscutti G (2017). Inception and utility of a renal replacement registry using administrative health data in north-East Italy. J Ren Care.

[15] RIDT (2017). Registro Italiano di Dialisi e Trapianto. Report 2014.

[16] Komenda P, Yu N, Leung S, Bernstein K, Blanchard J, Sood M (2015). Determination of the optimal case definition for the diagnosis of end-stage renal disease from administrative claims data in Manitoba. Canada C Open.

[17] Lok CE, Oliver MJ, Rothwell DM, Hux JE (2004). The growing volume of diabetes-related dialysis: a population based study. Nephrol Dial Transplant.

[18] Chowdhury TT, Hemmelgarn B, Parfrey PS, Barrett BJ (2015). Evidence-Based Decision-Making 6: Utilization of Administrative Databases for Health Services Research. Clinical Epidemiology: Practice and Methods. Second.

